# Recent Advances in Fluorine- and Silicon-Integrated Organic Solvent Nanofiltration Membranes for Non-Polar Solvent Separation

**DOI:** 10.3390/membranes16020057

**Published:** 2026-02-02

**Authors:** Shuo He, Weijia Song, Rongkai Che, Enlin Wang, Can Li, Baowei Su

**Affiliations:** 1 Key Laboratory of Marine Chemistry Theory and Technology, Ocean University of China, Ministry of Education, 238 Songling Road, Qingdao 266100, China; heshuo_ouc@126.com (S.H.); songweijia_ouc@126.com (W.S.); crk_ouc@126.com (R.C.); wangenlin_ouc@126.com (E.W.); 2 College of Chemistry & Chemical Engineering, Ocean University of China, 238 Songling Road, Qingdao 266100, China; 3 Singapore Membrane Technology Centre, Nanyang Environment and Water Research Institute, Nanyang Technological University, 1 Cleantech Loop, Singapore 637141, Singapore

**Keywords:** organic solvent nanofiltration (OSN), fluorinated and organosilicon-based membranes, non-polar solvent systems, separation mechanisms, practical applications

## Abstract

Organic solvent nanofiltration (OSN), also known as solvent-resistant nanofiltration (SRNF), is an emerging membrane-based separation technique capable of efficiently separating molecules in the 200–1000 Da range within organic media. It holds considerable promise for applications in organic solvent systems, which are prevalent in the petrochemical, pharmaceutical and food processing industries. While OSN has been extensively studied in polar solvent systems, increasing attention is now being directed toward its performance in non-polar environments, driven by their substantial practical demand and application potential. Fluorinated and organosilicon-based materials have emerged as key components in the fabrication of high-performance OSN membranes for separation in non-polar solvent environments due to their exceptional chemical, thermal, and mechanical stability. This review systematically summarizes recent advances in the design and fabrication of fluorinated and organosilicon-based composite OSN membranes. Key separation mechanisms are discussed, with particular focus on their roles in the recovery and reuse of homogeneous catalysts in chemical and pharmaceutical processes. Finally, future research directions are proposed to guide the continued development and industrial deployment of the fluorine- and silicon-based OSN membranes in non-polar solvent applications.

## 1. Introduction

Organic solvent nanofiltration (OSN), also referred to as solvent-resistant nanofiltration (SRNF), is a membrane-based separation technology designed for molecular-level separations in organic solvent environments. Unlike traditional nanofiltration technology, OSN membranes are engineered to maintain structural stability in strongly polar or non-polar organic media, enabling the separation of solutes in the 200–1000 Da range via precise pore-size sieving (under 2 nm) or dissolution–diffusion mechanisms [1,2,3]. The roots of OSN race back to the late 1990s, when scholars such as Linder filed a patent for OSN membranes in 1991 [2,3,4]. With the advancements of materials science and nanotechnology, OSN has seen significant development over the past decade. Its core advantage lies in separations without phase change [2,3,5,6,7], enabling energy savings of 80–90% compared to thermal processes such as distillation and extraction [2,3,8]. Therefore, OSN technology has great application potential in chemical engineering and petrochemicals, textiles, printing/dyeing, pharmaceuticals, and food processing [2,3,9,10,11,12], and is poised to play an increasingly important role in green chemistry and sustainable development.

**Figure 1:**
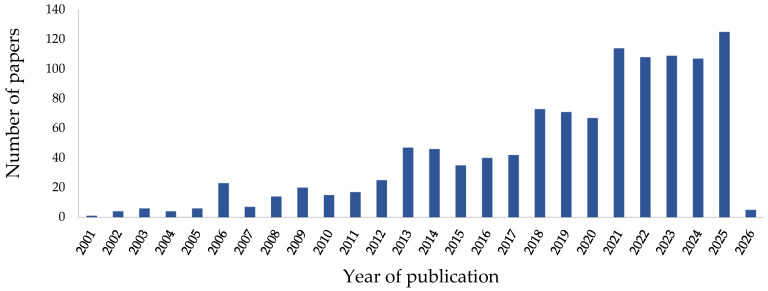
The number of papers on OSN/SRNF from 2001 to 2026.

**Figure 2:**
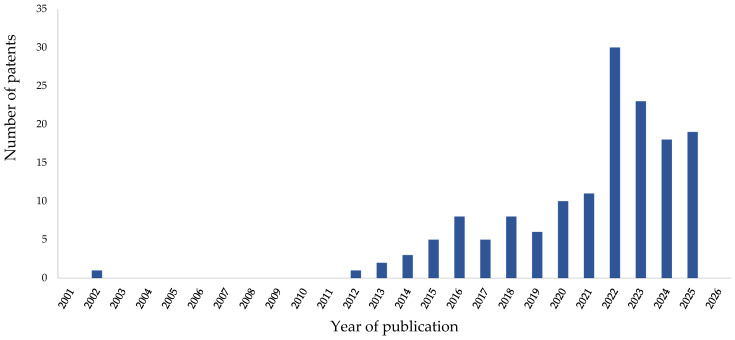
The number of patents on OSN/SRNF from 2001 to 2026.

As the core component of OSN technology, OSN membranes critically affect separation efficiency [2,3,13,14]. Since 2001, the number of publications and patents related to OSN membranes have grown rapidly, as reflected in Figure 1 and Figure 2 (data retrieved from SciFinder—CAS SciFinder Discovery Platform, using the keywords: “organic solvent nanofiltration” or “solvent-resistant nanofiltration”), especially in the recent five years.

Although OSN technology has achieved considerable progress, most research works were aiming at polar solvents (e.g., alcohols and ketones), and significant challenges remain for its implementation in non-polar solvent systems (such as n-hexane, toluene, cyclohexane) [2,3,15]. These difficulties primarily stem from the poor affinity and swelling effects of non-polar solvents on most polymeric membranes [2,3,16,17,18]. Conventional polymeric (polyamide and polyester) OSN membranes exhibit inherent hydrophilicity [2,3,19,20,21], which results in limited permeation of these non-polar solvents [2,3,22]. In the meantime, these membranes swell in non-polar solvent, which not only enlarges the membrane pores—thereby reducing rejection—but also leads to structural aging, performance degradation, and eventual membrane failure over time [2,3,23]. Therefore, increasing the hydrophobicity of OSN membranes has emerged as one of the most effective strategies to improve the permeance of non-polar solvents while maintaining solute rejection. Until now, the development of OSN membranes capable of long-term stable operation in non-polar solvent systems with both high non-polar solvent permeance and high selectivity remains a research frontier and a core technical difficulty in this field.

**Figure 3:**
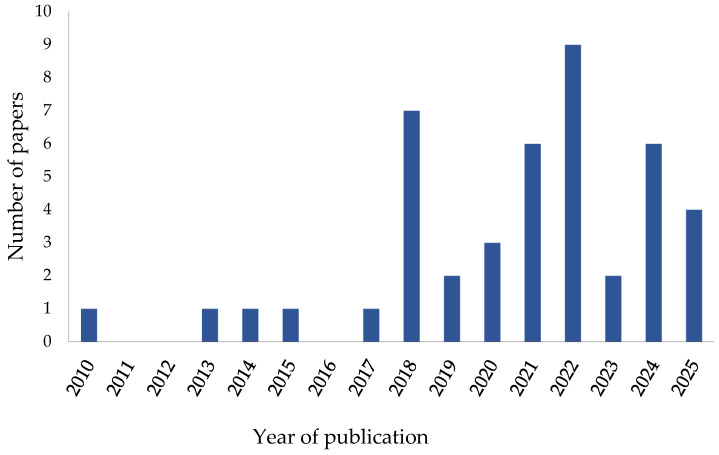
The number of papers on OSN membranes for non-polar solvent separations from 2010 to 2025.

To date, many studies have been focused on introducing hydrophobic functional groups as a key routine to improve the hydrophobicity of OSN membranes [2,3,24]. In recent years, significant progress has been made in OSN membranes for non-polar solvent systems, driven by advances in material design and membrane fabrication technologies. The growing research interest can be reflected by the increasing number of related publications, as shown in Figure 3.

During the past several years, researchers have developed many novel hydrophobic OSN membrane materials (such as fluorine compounds, silicon compounds, rigid microporous polymers, and amphiphilic materials) and have optimized OSN membranes fabrication processes including interfacial polymerization, surface grafting, and phase separation [2,3,25,26]. These innovations have not only addressed the low permeance challenge of non-polar solvents but also preserved the high solute retention capability of hydrophobic OSN membranes.

Fluorine compounds and silicon compounds have emerged as key materials with special physical and chemical properties in the advancement of hydrophobic OSN membranes [3,27]. Fluorine atoms, owing to their high electronegativity and small atomic radius, confer fluoropolymers with excellent chemical inertness, thermal stability, and hydrophobicity [3,28,29,30], making them highly suitable for hydrophobic OSN membrane fabrication. Silicon atoms, owing to their versatile valence and strong bonding properties, allow the formation of a stable siloxane bond (Si-O-Si) network [31], which endows organosilicon materials with superior mechanical strength and thermal resilience, thereby significantly enhancing the solvent resistance and separation performance of hydrophobic OSN membrane material [32].

This review systematically covers both the fabrication techniques involving fluorinated and organosilicon compounds for hydrophobic OSN membranes and the latest breakthroughs and future trends in their application to non-polar solvent systems. In addition, the performance of these hydrophobic OSN membrane materials in homogeneous catalyst separation and recovery is discussed, the separation mechanism is briefly introduced, and the future research directions are proposed to foster further development and practical deployment of fluorine- and silicon-based OSN membranes.

## 2. Fluorinated Compound-Based OSN Membranes

Fluorine-containing compounds occupy an important position in the field of membrane separation due to their unique physicochemical properties. Fluorine, the most electronegative element in the periodic table, forms carbon–fluorine (C-F) bonds with bond energies up to 485 kJ mol^−1^, which is much higher than those of C-H bonds (413 kJ mol^−1^) and C-C (347 kJ mol^−1^) bonds [33,34,35]. This exceptional bond strength imparts fluoropolymers with excellent chemical and thermal stability, as well as strong resistance to corrosion and swelling by non-polar solvents (e.g., toluene and n-hexane) [36,37]. As such, fluorinated materials have emerged as one of the core constituents for addressing the stability challenges in OSN membranes operating in non-polar solvent systems.

Fluorine-containing compounds can be broadly categorized into fluorinated polymer and fluorinated inorganic nanoparticles [38]. Fluoropolymers generally exhibit hydrophobic or even superhydrophobic behavior due to the low surface energy associated with C-F bonds [28,33], which is beneficial for reducing solute adsorption and contamination on the membrane surfaces. This characteristics make fluoropolymers highly suitable for use in non-polar solvent systems, where they provide outstanding resistance to swelling or degradation by non-polar solvents. Common fluoropolymers include polyvinylidene fluoride (PVDF) [39], polytetrafluoroethylene (PTFE), Teflon AF2400 [40], and perfluorosulfonic acid resin Nafion. Among them, PVDF demonstrates superior wear resistance, impact resistance, and chemical corrosion resistance due to its low surface energy, which also contributes to its strong hydrophobicity—commercial PVDF porous membranes commonly show a water contact angle of around 124° [41]. In addition, PVDF has excellent film-forming capability. Another well-known fluoropolymer, PTFE, often dubbed the “king of plastics”, offers exceptional chemical inertness and minimal reactivity toward most chemical substances also due to its low surface energy. Teflon AF2400, which is synthesized from perfluoro-2,2-dimethyl-1,3-dioxole (PDD or PDDD) and tetrafluoroethylene (TFE), is an amorphous, glassy perfluoropolymer with a high fractional free volume (33%), strong hydrophobicity, and exceptional chemical and thermal stability. To date, all these fluoropolymers have been extensively investigated for application as hydrophobic OSN membrane materials. Fluoropolymers could enable rigid and hydrophobic fluorinated networks that resist solvent-induced swelling and preserve pore integrity. Additionally, the introduction of fluorine groups reduces the membrane affinity for polar solutes (e.g., dyes) while promoting the permeation of non-polar solvents, thereby enhancing the separation selectivity through the regulated affinity difference between the membrane and the two phases.

Fluorinated inorganic nanoparticles, inorganic nanomaterials functionalized with fluorine atoms or fluorine-containing groups via surface modification or bulk doping, can be incorporated into the polymer matrix and can also effectively tailor the material’s surface properties and transport behavior. The introduction of fluorinated inorganic nanoparticles reduces the membrane affinity for polar solutes (e.g., dyes) while promoting the permeation of non-polar solvents owing to their low-surface-energy interfaces. Meanwhile, these nanoparticles enhance non-polar solvent permeance and selectivity by creating precise nanoscale channels, while their chemically inert fluorinated surface improves solvent resistance [42,43].

### 2.1. Fluoropolymers as Substrates of OSN Membranes

Fluoropolymers can serve as a substrate of OSN membranes via phase inversion. PVDF and PTFE are usually used as substrates of hydrophobic OSN membranes. For example, Matthias et al. [44] fabricated PVDF membranes through phase inversion and subsequently cross-linked it with p-phenylenediamine (XDA) under alkaline conditions, followed by activation with dimethylformamide (DMF). The optimal OSN membrane achieved a toluene permeance of 9.4 L m^−2^ h^−1^ MPa^−1^ and a rose bengal (RB) rejection of 98%, indicating its excellent performance in harsh non-polar solvent environments. Chen et al. [29] fabricated a kind of PVDF UF membrane via the immersion precipitation phase inversion method, then cross-linked it using potassium persulfate as the initiator and polyvinylpyrrolidone (PVP) as the cross-linking agent. The cross-linked membrane exhibited improved solvent stability, increased dye retention, and reduced solvent permeation flux. Reported rejection of dye RB in solvents such as isopropylamine (IPA) and 1-aminocyclopropanecarboxylic acid (ACC) exceeded 90%, with the rejection in tetrahydrofuran (THF) reaching 95.2%.

### 2.2. Fluorinated Compounds as Functional Monomer

Fluorinated compounds can be used as a functional monomer for surface modification via grafting, or used as a reactive monomer for constructing fluorinated cross-linked polyamide networks of the functional layer of hydrophobic OSN membranes via interfacial polymerization. Using fluorinated compounds as one of the reactive monomers allows their permanent integration into the backbone or side chains of the cross-linked network in the form of covalent bonds [45].

#### 2.2.1. Fluorinated Compounds as Surface Modifying Agent

The strong electronegativity, small atomic radius, and low polarity of fluorine atoms influence the polymerization process and thus regulate the cross-linking density, free volume, and surface energy of the polyamide network, ultimately yielding enhanced solvent resistance and tailored selectivity [46].

**Figure 4:**
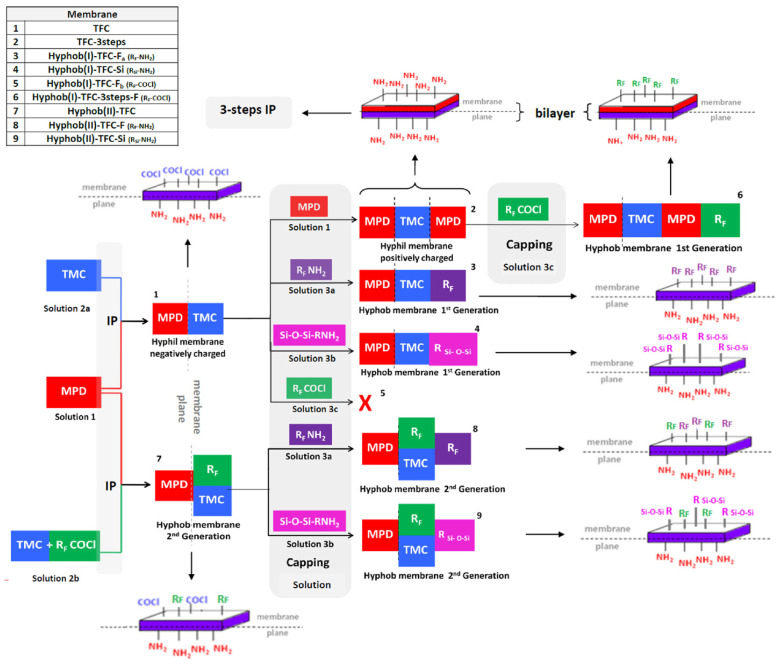
Strategies for hydrophobic TFC membrane formation. Reprinted with permission from Ref. [47]. Copyright 2013, Elsevier.

Fluorinated compounds have been initially used as surface modifying agent for improving hydrophobicity. For example, Jimenez Solomon et al. [47] chemically modified an MPD/TMC polyamide membrane’s surface by capping the unreacted acyl chloride groups with hydrophobic fluorinated monomers containing amino groups, and used fluorine amine as a co-monomer to increase permeability in non-polar solvents, as shown in Routine 3 presented in Figure 4. For Nanofiltration of a feed solution comprising polystyrene oligomers dissolved in toluene, the fluorinated OSN membrane shows the highest flux in toluene (51 L m^−2^ h^−1^), more than five times that of the baseline TFC, which is only 9 L m^−2^ h^−1^ at 3.0 MPa and 30 °C, without sacrificing solute rejection. They also carried out a secondary interfacial polymerization between MPD and fluorinated acyl chloride on the nascent MPD/TMC polyamide membrane, as shown in Routine 6 presented in Figure 4. The formed OSN membrane has a toluene flux of 15 L m^−2^ h^−1^, three times higher than that of the baseline TFC, which is only 5 L m^−2^ h^−1^ at 3.0 MPa and 30 °C, accompanied with an apparently increased solute rejection from about 90% to about 93% at MW of 300 Da. They called these kinds of OSN membranes the first-generation hydrophobic membrane.

#### 2.2.2. Fluorinated Compounds as Oil-Phase Monomer of Interfacial Polymerization

Apart from their use as a surface modifying agent, fluorinated compounds can be used as oil-phase monomers or co-monomers, to react with diamines (e.g., m-phenylenediamine, MPD) from the aqueous phase to form the fluorinated polyamide backbones. The strong electronegativity, small atomic radius, and low polarity of fluorine atoms influence the polymerization process and thus regulate the cross-linking density and surface energy of the polyamide network, which helps to modulate the network structure, increase oleophobicity, and expand the free volume, ultimately yielding enhanced solvent resistance and tailored selectivity [46]. For example, in the same work of Jimenez Solomon et al. [47], they also incorporated hydrophobic fluoro-monoacyl chloride with TMC in the organic phase during the IP reaction to hinder cross-linking, resulting in a more open polymer network, which was the most effective way to enhance solvent flux, as shown in Routine 7 presented in Figure 4. The fluorinated OSN membrane shows the even higher flux of toluene (85.7 L m^−2^ h^−1^) as compared with the baseline TFC (9.4 L m^−2^ h^−1^) at 3.0 MPa and 30 °C, but with a slightly decreased rejection, with a PS-based MWCO of <600 Da. They further carried out a surface modification on this membrane with amine, as shown in Routine 8 presented in Figure 4. The fluorinated OSN membrane shows the even higher flux of toluene (109.3 L m^−2^ h^−1^) as compared with the baseline TFC (9.4 L m^−2^ h^−1^) at 3.0 MPa and 30 °C, but with an even slightly decreased rejection, with a PS-based MWCO of >600 Da.

**Figure 5:**
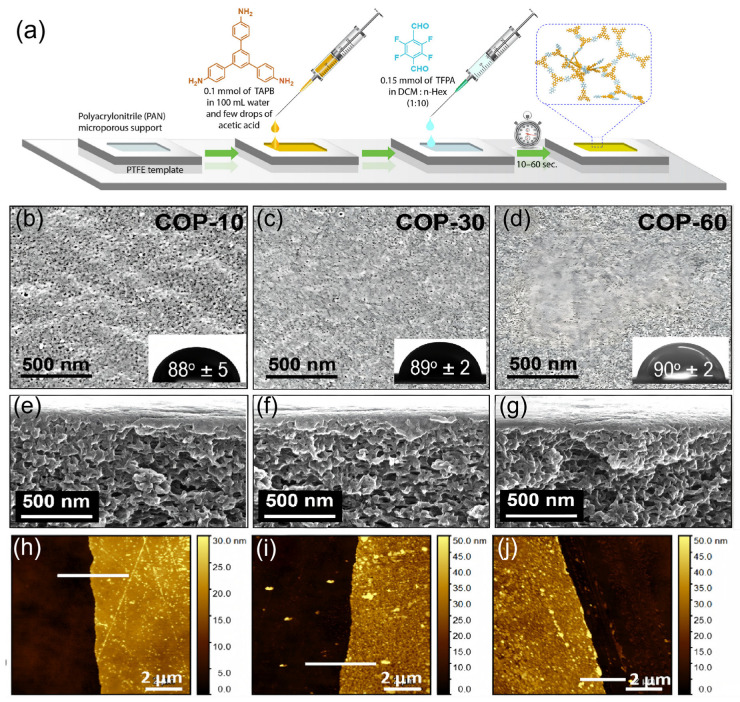
Schematic of the rapid fabrication of covalent organic polymer membranes via an interfacial polymerization reaction between 0.1 mmol TAPB and 0.15 mmol (TFPA) monomers (**a**), the template was poly(tetrafluoro ethylene)-based (PTFE). (**b**–**g**) Morphological characterizations of covalent organic polymer (COP) membranes prepared with various reaction durations: (**b**,**e**) 10 s, (**c**,**f**) 30 s, (**d**,**g**) 60 s. Scanning electron micrographs: surface view (**b**–**d**) where insets show contact angle measurements, and (**e**–**g**) cross-sections; (**h**–**j**) thickness profile. Reprinted with permission from Ref. [48]. Copyright 2022, Elsevier.

Aldurai et al. [48] implemented ultra-rapid polymerization reaction (10–60 s) between TAPB (an amino monomer) and TFPA (a fluorinated aldehyde monomer) on the PAN substrate to form a fluorinated OSN membrane (Figure 5). The resulting membrane was ultra-thin (14–23 nm), hydrophobic (water contact angle of approximately 90°), and thermally stable (>400 °C), and had a smooth, defect-free, and crack-free surface (Figure 5b–g). The membrane achieved a toluene permeance of 110 L m^−2^ h^−1^ MPa^−1^ and >95% rejection of Congo red (687 g mol^−1^). The OSN membrane was subjected to continuous solvent filtration tests over 24 h in pure acetone and toluene, respectively, and retained essentially unchanged permeance, indicating negligible performance decay. During a five-day filtration experiment using Rhodanile Blue/toluene as feed, the membrane maintained a Rhodanile Blue rejection of approximately 97.5% and a stable toluene permeance in the range of 100 to 110 L m^−2^ h^−1^ MPa^−1^ throughout the test period, which demonstrates its excellent long-term stability in non-polar solvents.

#### 2.2.3. Fluorinated Compounds as Aqueous-Phase Monomer of Interfacial Polymerization

**Figure 6:**
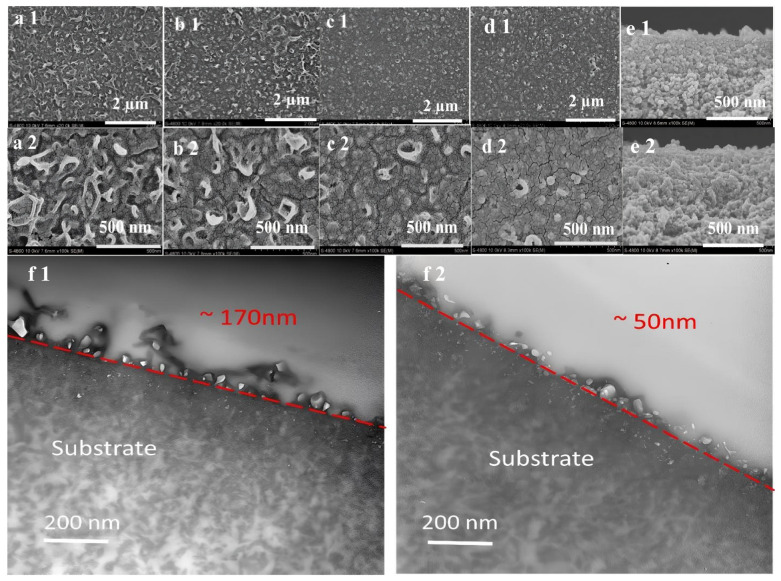
Surface SEM images of the (PA/PI)xa (**a1**,**a2**), (FPA-0.2/PI)xa (**b1**,**b2**), (FPA-0.5/PI)xa (**c1**,**c2**) and (FPA-1.0/PI)xa (**d1**,**d2**) OSN membranes, and the cross-sectional SEM images of the (PA/PI)xa (**e1**) and (FPA-0.5/PI)xa (**e2**) OSN membranes. Cross-sectional TEM images of the (PA/PI)xa (**f1**), and (FPA-0.5/PI)xa (**f2**) OSN membranes. Reprinted with permission from Ref. [49]. Copyright 2019, Elsevier.

Fluorinated compounds can be used as aqueous-phase monomers, to react with acyl chlorides (such as trimesoyl chloride, TMC) in the organic phase, resulting in fluorine atoms being directly attached to the polymer backbone or side chains. For example, Guo et al. [49] employed a polyimide (PI) ultrafiltration support and conducted interfacial polymerization between MPD and 5-trifluoromethyl-1,3-phenylenediamine (TFMPD) in the aqueous phase and TMC in the oil phase. They further performed hexanediamine (HDA) chemical cross-linking and dimethylformamide (DMF) solvent activation and prepared a fluorine-containing TFC OSN membrane. The incorporation of TFMPD resulted in a smoother and thinner separation layer compared to the membranes fabricated without TFMPD (Figure 6). The water contact angle increased from 64.1° to 82.9°, indicating the reduced surface energy and decreased hydrophilicity. At the optimal TFMPD/MPD mass ratio of 0.5, the ethyl acetate permeance reached ~25 L m^−2^ h^−1^ MPa^−1^. However, since TFMPD was involved in the aqueous monomer solution, the membrane surface is covered by TMC rather than TFMPD, leaving residual acyl chloride groups that hydrolyzed to carboxylic acid groups on the IP cortex surface. Consequently, the membrane surface remained relatively hydrophilic rather than fully hydrophobic. A similar research work was carried out by Xu et al. [25] who used fluorinated diamine 4,4′-oxybis(3-(trifluoromethyl)aniline (OTFA) and TMC as reactants to perform interfacial polymerization on solvent-resistant polyketone (PK) substrates and fabricated a fluorinated composite OSN membrane featuring a thicker yet highly hydrophobic polyamide layer and a high surface fluorine content. This OSN membrane demonstrated significant permeance to non-polar solvents, with a toluene permeance of 97.5 L m^−2^ h^−1^ MPa^−1^ and a 96% rejection for Irganox 3114 (an antioxidant with molecular weight of 784 Da).

**Figure 7:**
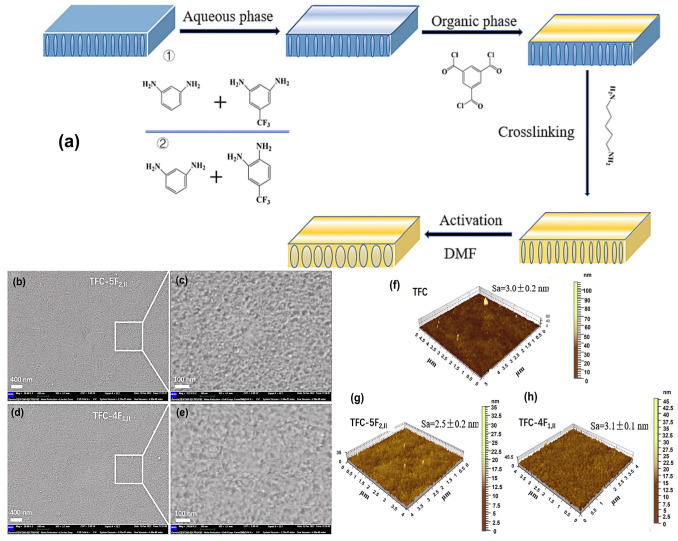
Preparation process of the fluorinated polyamide TFC OSN membrane (**a**). Surface SEM images of the (**b**,**c**) TFC-5F_2,II_ and TFC-4F_1,II_ (**d**,**e**) membranes, AFM images of membrane surfaces of the (**f**) TFC, (**g**) TFC-5F_2,II_, (**h**)TFC-4F_1,II_ membranes. Reprinted with permission from Ref. [51]. Copyright 2024, Elsevier.

However, it should be noted that the inherently low aqueous solubility of fluorinated diamines (e.g., TFMPD, OTFA) presents a challenge for achieving a homogeneous aqueous-phase solution [50]. This limitation largely restricts the application of fluorinated polymers in OSN membranes. The use of ultra-low monomer concentrations could avoid the problem of inherently low aqueous solubility of fluorinated diamines. Chen et al. [51] employed ultra-low concentrations (0~0.05 wt%) of fluorinated diamines (5TFMPD or 4TFOPD) as a co-monomer with MPD in the aqueous phase (fixing overall monomer concentration of 0.05 wt%) for interfacial polymerization with TMC on a PI ultrafiltration substrate. Ultra-low monomer concentrations help mitigate aggregation and facilitate more uniform diffusion of amine molecules toward the organic interface, thereby promoting the formation of a defect-free selective layer. In this way, they obtained two types of smooth, ultra-thin fluorinated polyamide TFC OSN membranes (TFC-5F_2,II_ and TFC-4F_1,II_) with a typical nodular surface structure (as depicted in Figure 7) and a surface roughness values of 2.5 nm and 3.1 nm, respectively, and an increased water contact angle from 40.8° of the baseline to 86.4° and 83.1°, respectively, reflecting significantly enhanced hydrophobicity via the introduction of the -CF_3_ groups. The fluorinated OSN membranes have superior separation peformance, with the ethanol permeance of 73.9 L m^−2^ h^−1^ MPa^−1^ and 60.9 L m^−2^ h^−1^ MPa^−1^ with the RDB rejection of 98.5% and 99.2%, respectively, for the Rhodamine B (RDB)/ethanol system. In the non-polar ethyl acetate system, they achieved permeance of 209.4 L m^−2^ h^−1^ MPa^−1^ and 117.0 L m^−2^ h^−1^ MPa^−1^, with catalyst (Jacobson) rejection of 98.2% and 98.8%, respectively. These results highlight the high flux capability of the fluorinated OSN membranes for both polar and weak polar solvents and underscore their considerable potential for separation and purification in diverse application domains.

The use of ionic liquids could also avoid the problem of inherently low aqueous solubility of fluorinated diamines. Ionic liquids (ILs)—organic salts that are molten at ambient temperature—offer a promising alternative: they exhibit extremely low vapor pressure, high chemical stability, strong solubility for aromatic amines, and tunable physical–chemical properties [52,53]. By establishing an anhydrous interface between the ionic liquid and an alkane, the hydrolysis of acyl chloride during interfacial polymerization can be completely avoided [54]. For example, Ma et al. [50] replaced the conventional aqueous phase with an ionic liquid/water mixed solvent and dissolved a combination of fluorinated monomers (hexafluorobisphenol A, BPAF) and bisphenol A (BPA) therein, and then performed the interfacial polymerization with TMC on a PAN support. In this way, they successfully produced a fluorinated polyarylate TFC membrane. Then they further cross-linked it using hydrazine hydrate to improve layer adhesion. The resultant fluorine-containing TFC membrane had a pure toluene permeance of 110 L m^−2^ h^−1^ MPa^−1^. The high viscosity of the ionic liquids and the addition of fluorine-containing monomers slowed the polymerization kinetics, resulting in a thinner selective layer and significantly improved hydrophobicity and solvent resistance.

#### 2.2.4. Fluorinated Compounds as Both Aqueous- and Oil-Phase Monomers

In addition, fluorinated compounds can also be served both as aqueous- and oil-phase reactive monomers or co-monomers within the same interfacial polymerization process. For example, Alduraiei et al. [26] used 5-trifluoromethyl-1,3-phenylenediamine (TFMP) in the aqueous phase and a mixture of trimesoyl chloride (TMC) and a self-made fluorinated compound, HFBC (4,4′-hexafluoroisopropylidene bis(benzoyl chloride)), in the organic phase as a co-monomer to perform the interfacial polymerization on a polyacrylonitrile (PAN) ultrafiltration support membrane, and fabricated a fluorinated polyamide thin-film composite (TFC) OSN membrane. The resulting membrane exhibited a high fluorine content of up to 26.3% and substantial hydrophobicity (water contact angle of approximately 100°). It achieved a rejection of 87% for hexaphenylbenzene (MW = 534 g mol^−1^) and a toluene permeance of 100 L m^−2^ h^−1^ MPa^−1^, and demonstrated excellent stable performance for 5 days of continuous operation.

In summary, employing fluorinated compounds as functional monomers in interfacial polymerization allows for the direct incorporation of fluorine into the polyamide backbone, yielding membranes with enhanced hydrophobicity (water contact angles higher than 80°), good solvent permeance, and improved solute rejection. However, a challenge still remains due the low aqueous solubility of many fluorinated amines. Nevertheless, this can be partly alleviated through co-solvent assistance [55,56] or ionic-liquid-based reaction media.

### 2.3. Fluoropolymers as Functional Coating Layer

The application of fluoropolymers as a functional coating layer of OSN membranes does not rely on covalent incorporation into the polymer backbone; instead, they are physically coated onto the substrate surface.

**Figure 8:**
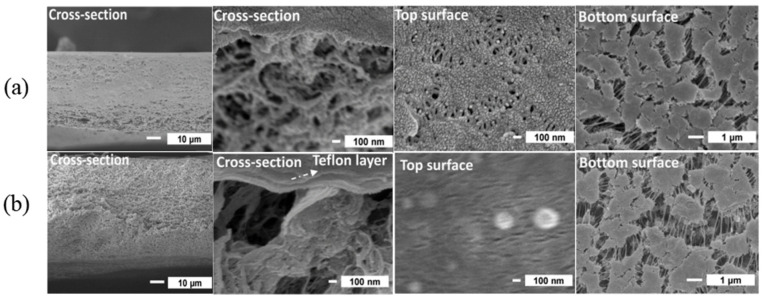
The membrane morphology of (**a**) PE 5 nm and (**b**) Teflon/PE membrane prepared with 0.5 wt% Teflon concentration in 0% RH. Reprinted with permission from Ref. [59]. Copyright 2020, Elsevier.

Fluorinated polymers—typically linear or branched fluorinated polymers—interact with the primary membrane material (e.g., polyimide) [57,58], forming low-surface-energy domains or promoting pore architectures that facilitate solvent transport, thereby enhancing membrane performance in organic solvent environments. For example, Teflon AF2400 is usually used as a functional layer of hydrophobic OSN membranes. Shi et al. [59] coated a Teflon AF2400 solution onto a porous polyethylene (PE) support and formed an ultra-thin selective layer by adjusting coating concentration and drying relative humidity. The resulting Teflon/PE membrane featured a ~100 nm Teflon layer (Figure 8), and exhibited high permeance to low-polarity solvents such as hexane and toluene, and impermeance to strongly polar solvents like DMF and DMSO. Table 1 summarizes the physical and chemical properties of eight commonly used solvents, including the Hansen solubility parameters obtained from the HSPiP software. The preferential transport of hydrophobic solvents is primarily attributed to the strong hydrophobicity of Teflon AF2400, whose solubility parameters closely match those of weakly polar solvents. Under optimal conditions (0.5 wt% Teflon and 0% humidity drying), the membrane exhibited permeabilities of 33–86 L m^−2^ h^−1^ MPa^−1^ for non-polar solvents including n-hexane, toluene, and ethyl acetate, and in a 20 wt% oil/n-hexane system it delivered a triglyceride (800~900 Da) rejection of 99.5% with the n-hexane permeance of 14.5 L m^−2^ h^−1^ MPa^−1^. Li et al. [40] employed a commercial AF2400/PTFE membrane to filtrate hexane solution of 32 wt% soybean oil as feed at 2.0 MPa and 30 °C. The permeate flux varied between 8 and 11 L m^−2^ h^−1^ MPa^−1^, and the rejection of the soybean oil consistently exceeded 98%.

**Figure 9:**
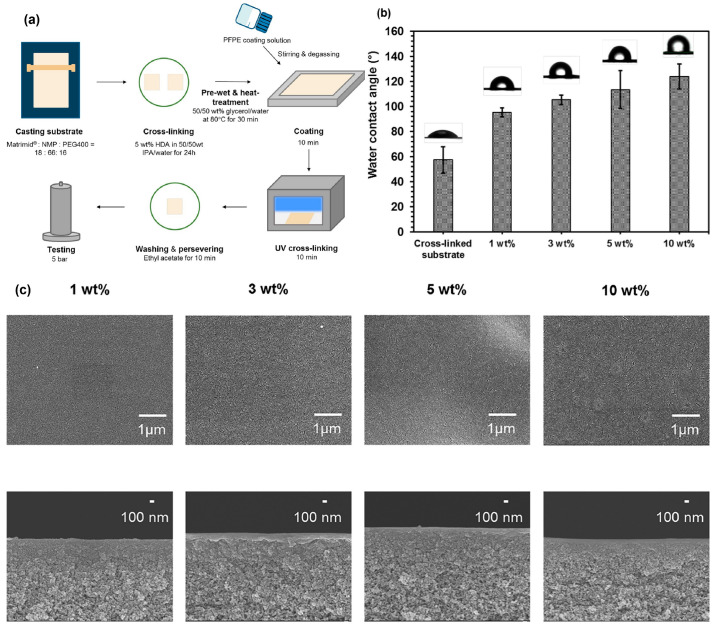
Schematic diagram for the fabrication process of PFPE-coated membranes (**a**), water contact angles of the cross-linked substrate and PFPE-coated membranes as a function of PFPE coating concentration (**b**), FESEM images of the top and cross-section morphologies of PFPE-coated membranes (**c**). Reprinted with permission from Ref. [60]. Copyright 2019, American Chemical Society.

PVDF and PTFE can also be used as the functional coating layer of hydrophobic OSN membranes. For example, Li et al. [60] coated a kind of UV-curable fluorinated perfluoropolyether (PFPE, Fluorolink AD1700) on a cross-linked Matrimid^®^ polyimide support, and then UV-cured the film to impart high hydrophobicity and chemical stability. They obtained an optimized PFPE OSN membrane which exhibits a beta-carotene (536.9 Da) rejection rate of 94.2 ± 3.4% for the non-polar system of beta-carotene/n-hexane, with a permeance of 7.7 ± 0.8 L m^−2^ h^−1^ MPa^−1^, as illustrated in Figure 9a.

### 2.4. Fluorinated Inorganic Nanoparticles as Functional Additives

Fluorinated inorganic nanoparticles have been applied as functional additives which are physically incorporated into the separation layer and retained within the polymer matrix of OSN membranes through non-covalent interactions such as van der Waals forces or hydrogen bonding. Incorporating fluorinated inorganic nanoparticles into the polymer matrix can also effectively tailor the material’s surface properties and transport behavior. Common incorporation techniques, such as phase inversion and interfacial polymerization, can be used to embed fluorinated inorganic nanoparticles into the polymer matrix. These nanoparticles enhance solvent permeance and selectivity by creating precise nanoscale channels and low-surface-energy interfaces, while their chemically inert fluorinated surface improves solvent resistance [42,43].

For example, Mohamed et al. [61] fabricated hydrophobic poly(divinylbenzene-co-perfluorodecyl acrylate) (poly(DVB-co-PFDA)) nanoparticles via free radical precipitation polymerization and incorporated them into the polyamide layer of a thin-film composite (TFC) membrane via interfacial polymerization. The resulting thin-film nanocomposite (TFN) membrane exhibits a permeance to pure toluene of approximately 64 L m^−2^ h^−1^ MPa^−1^, which is significantly higher than that of the unmodified TFC membrane. The enhancement was attributed to the increased surface roughness and hydrophobicity induced by the nanoparticles, which improved the membrane’s affinity for non-polar solvents.

The physical blending of fluorinated nanoparticles offers a versatile route to enhance membrane hydrophobicity and solvent resistance without requiring covalent bonding. These additives create low-surface-energy domains and, in some cases, may introduce additional nano-channels for solvent transport, thereby enhancing permeance for non-polar solvents while maintaining high rejections (>95%) for oils or dyes. However, attention must be paid to the compatibility between the additive and the polymer matrix to avoid defects that could compromise membrane selectivity. While the incorporation of fluorinated inorganic nanoparticles can enhance solvent permeance and surface properties, it may also introduce non-selective voids or interfacial defects if the nanoparticles are poorly dispersed or exhibit poor compatible with the polymer matrix. Such defects can compromise membrane selectivity by creating unintended transport pathways or increasing local pore sizes, thus elevating solvent flux at the expense of solute rejection. To mitigate this issue, various strategies have been adopted. Surface modification of nanoparticles (e.g., silanization) can improve interfacial compatibility and dispersion within the polyamide matrix. In situ growth techniques, such as sol–gel methods, allow better integration of nanoparticles during membrane formation. Additionally, post-treatment processes, including thermal curing or chemical cross-linking, are employed to seal interfacial voids and reinforce the selective layer.

### 2.5. Summary of Fluorinated OSN Membranes

To summarize, fluorinated compounds can be used as a substrate, a skin layer via interfacial polymeriztion and coating, or via incorporation as nanofillers. The comparison of the performances of fluorinated thin-film composite OSN membrane by different fabrication methods is shown in Table 2, which highlights that the introduction of fluorinated compounds generally enables the fabrication of membranes with high flux and high rejection rates. Among these, membranes prepared via interfacial polymerization using fluorinated monomers exhibit higher permeability to non-polar solvents while maintaining excellent solute rejection (>95%). The summary of fluorine functional groups and surface energy in fluorinated OSN membranes is listed in Table 3, showing that the introduction of -CF_3_, -CF_2_-, and other fluorinated groups significantly increases water contact angles and reduces surface energy, thereby enhancing membrane hydrophobicity and compatibility with non-polar solvents.

In non-polar solvent systems, the dominant separation mechanism of fluorinated membranes is mainly size sieving, enabled by rigid and hydrophobic fluorinated networks that resist solvent-induced swelling and preserving pore integrity. Additionally, the introduction of fluorine groups reduces the membrane’s affinity for polar solutes (e.g., dyes) while promoting non-polar solvent permeation, thereby enhancing selectivity through a combined solubility-diffusion effect.

## 3. Silicon-Containing OSN Membranes

Silicon compounds have long been widely used in membrane separation due to their highly tunable chemical structure and controllable surface properties. Silicon, situated in Group IV of the Periodic Table, forms Si-O bonds with bond energies up to 460 kJ mol^−1^ and a relatively long bond length, endowing organosilicon materials with exceptional thermal stability and mechanical robustness [65,66]. Organic inorganic hybrids—such as siloxanes, silica sols, and silicones—combine the flexibility of organic moieties with the structural integrity of inorganic frameworks, thereby offering excellent film-forming ability and solvent resistance for OSN membrane fabrication. Furthermore, grafting of silane coupling agents introduces organosilicon groups to the membrane surface, lowering surface energy and significantly improving wettability of non-polar solvents [67].

The most common silicon-based materials used in OSN membranes include polydimethylsiloxane (PDMS), silicon dioxide (SiO_2_) and its composites, and polydisilsesquioxane (PSSQ). These materials find diverse applications in solvent resistant nanofiltration membranes—as a functional matrix, nanofillers, cross-linking agents, and surface modifiers—thereby enhancing key performance metrics under challenging solvent conditions.

### 3.1. Silicon Polymer as Functional Selective Layer Matrix

Silicon-based materials—particularly polydimethylsiloxane (PDMS)—have gained prominence in the fabrication of the functional selective layer matrix of TFC OSN membranes. As a representative organosilicon polymer, PDMS offers excellent chemical resistance, low surface energy, and good flexibility, making it highly suitable for forming the selective layer. This approach leverages the intrinsic hydrophobic and organophilic properties of siloxanes to create separation layers specifically engineered for non-polar systems.

The most straightforward method to create a silicon-based selective layer is through surface coating. This method involves immersing a prefabricated porous support into a silicone-containing prepolymer solution (e.g., PDMS), followed by curing to form a continuous hydrophobic silicone layer on its surface. The process is straightforward, and the layer thickness and morphology are readily tunable. Common base supports in this context include PAN (molecular weight cut-off, MWCO, ranging from 20,000 to 100,000), PVDF, and other porous membranes such as polysulfone (PSf) [2]. For example, Suleman et al. [68] prepared asymmetric PSf membranes via the phase separation method and subsequently immersed them in PDMS coating solution. The PDMS deposition significantly improved the surface hydrophobicity, increasing the contact angle from 53.18° for the unmodified PSf to 71° for PSF/PDMS300. The enhanced hydrophobic surface suggests vast potential resistance to polar solvent infiltration.

However, although surface coating is simple and easy to implement, PDMS membranes undergo substantial swelling in non-polar solvents such as toluene [69,70], which leads to a significant decline in selectivity and limits their applicability in OSN. Meanwhile, the bonding strength between the coating and the substrate is often limited by physical adsorption.

### 3.2. Silicon Compounds as Functional Monomer

**Figure 10:**
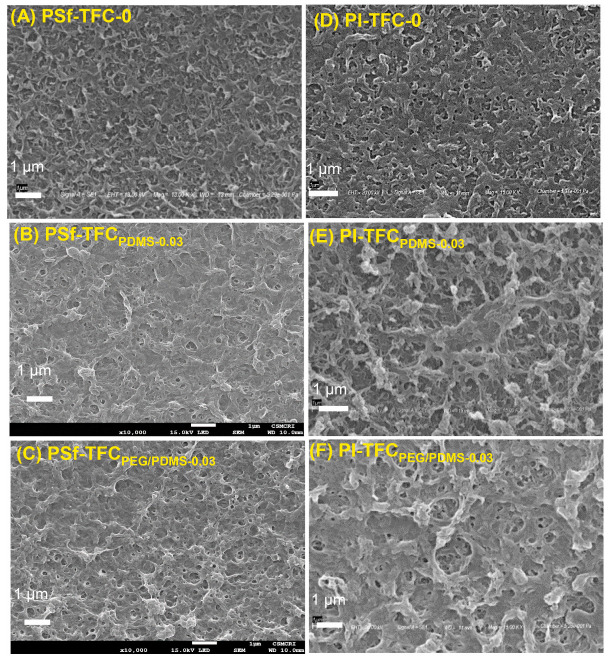
SEM images of pristine (**A**,**D**) and representative hydrophobic (**B**,**E**) and amphiphilic (**C**,**F**) membranes. Reprinted with permission from Ref. [22]. Copyright 2021, Elsevier.

**Scheme 1:**
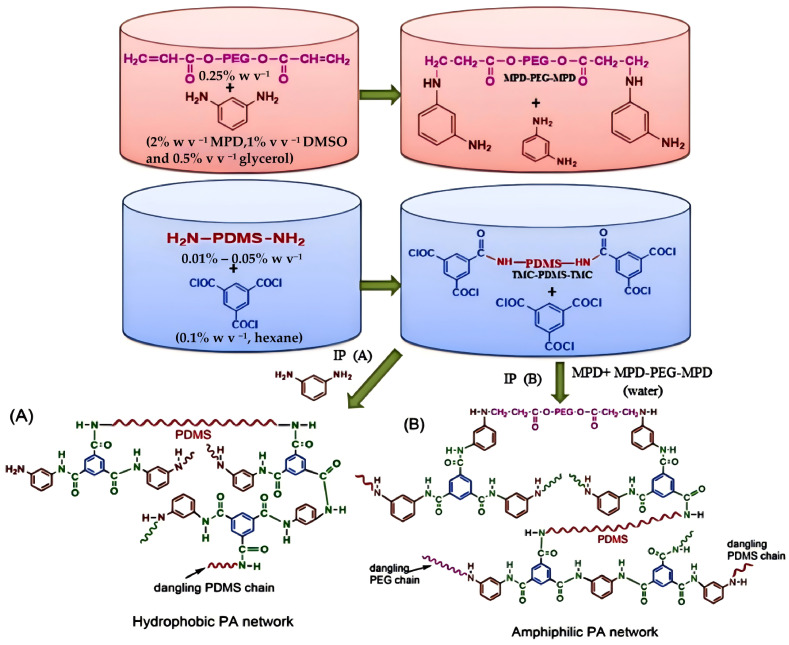
Strategy for the preparation of hydrophobic and amphiphilic PA TFC membranes by the interfacial polymerization between (**A**) MPD and TMC-PDMS-TMC + TMC (hydrophobic) and (**B**) MPD-PEG-MPD + MPD and TMC-PDMS-TMC + TMC (amphiphilic), respectively. Reprinted with permission from Ref. [22]. Copyright 2021, Elsevier.

**Figure 11:**
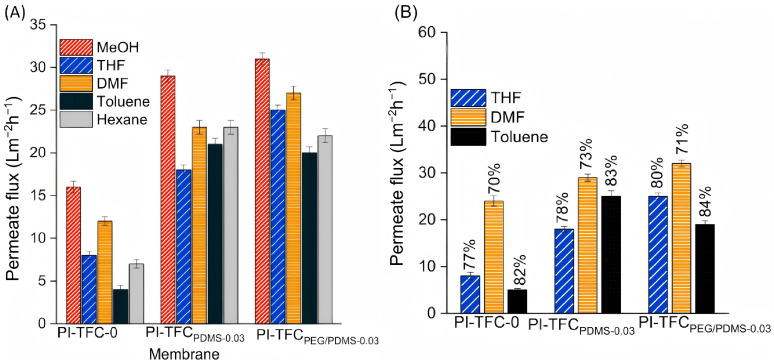
(**A**) Permeate fluxes (dead-end system) of solvents through representative PI-TFC-0, PI-TFC_PDMS-0.03_ and P-TFC_PEG/PDMS-0.03_ membranes. (**B**) Permeate flux (crossflow filtration) of representative solvents containing PEG marker (600 g mol^−1^, rejection values are indicated). The filtration operations were performed at an applied pressure of 27.5 bar. Reprinted with permission from Ref. [22]. Copyright 2021, Elsevier.

To achieve a more stable silicon-based functional layer, researchers have further introduced silicon-containing monomers directly into the interfacial polymerization process. Silicon-containing monomers can be directly added to the aqueous or organic phase, allowing them to participate in the reaction and become covalently embedded within the polyamide network, thereby constructing a hybrid separation layer that combines high hydrophobicity with solvent stability. Building on the strategy of introducing a single type of silicon-containing monomer, a more systematic design involves incorporating complementary silicon-based precursors into both the aqueous and organic phases simultaneously, enabling them to undergo in situ cross-linking during interfacial polymerization. This method constructs a hybrid separation layer characterized by a dense structure and a smooth surface, which contributes to a more integrated architecture and finely tunable performance. Trivedi et al. [22] constructed hydrophobic and amphiphilic polyamide separation layers on both polysulfone (PSf) and cross-linked polyimide (PI) supports by introducing TMC-capped PDMS (TMC-PDMS-TMC) into TMC/n-hexane solution to serve as the organic phase co-monomers to react with MPD via interfacial polymerization, as shown in Routine A in Scheme 1. Further, they followed the above procedure with the exception of introducing MPD-terminated polyethylene glycol (MPD-PEG-MPD) into the aqueous MPD phase and constructed amphiphilic polyamide separation layers, as shown in Routine B in Scheme 1. The in situ grafting of PDMS chains significantly enhanced the membrane’s hydrophobicity, affinity for non-polar solvents, and overall solvent resistance. As shown in Figure 10, the modified membranes (B/C and E/F) exhibit denser, smoother surfaces with no obvious exposed pores. Accordingly, the water contact angle increased from 50° for the pristine PI-TFC-0 membrane to 101° for the hydrophobic PI-TFC_PDMS-0.03_ membrane. In the separation of a non-polar PEG600/toluene system at 2.75 MPa, the PI-TFC-PDMS-0.03 membrane achieved a PEG600 retention rate of approximately 84% while maintaining a pure toluene flux of 5.5–6.5 L m^−2^ h^−1^ MPa^−1^ (Figure 11).

### 3.3. Silicon-Based Cross-Linking Agent

Unlike nanofillers, silicon-based compounds are also widely used as cross-linkers to enhance the performance of OSN membranes. By forming a stable three-dimensional network structure through siloxane bonds (Si-O-Si) or other chemical bonds, these cross-linkers significantly improve membrane solvent resistance and mechanical strength while effectively tuning the separation performance.

**Figure 12:**
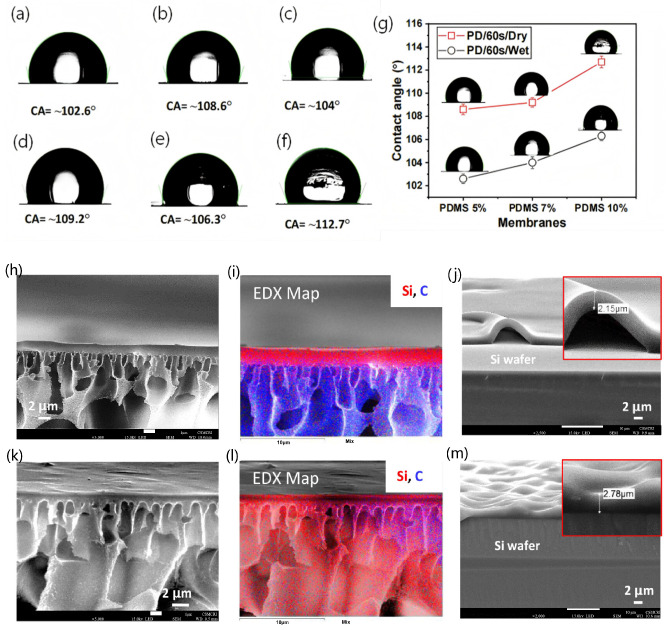
Water contact angles of the PMS-PANMA membranes, (**a**) PD/5%/60 s/Wet, (**b**) PD/5%/60 s/Dry, (**c**) PD/7%/60 s/Wet, (**d**) PD/7%/60 s/Dry, (**e**) PD/10%/60 s/Wet, (**f**) PD/10%/60 s/Dry. (**g**) Depiction of increase in the water contact angles for the membranes prepared with increase in initial pre-polymer concentration. Cross-sectional morphological analysis of the PMS-PANMA TFC membranes PD/10%/60 s/Wet (**h**) and PD/10%/60 s/Dry (**k**) by SEM. Si and C elemental mapping by SEM-EDX of the PMS-PANMA TFC membranes PD/10%/60 s/Wet (**i**) and PD/10%/60 s/Dry (**l**). Thickness analysis of the PMS layer by SEM after it was transferred onto a silicon wafer by separation from the TFC membranes PD/10%/60 s/Wet (**j**) and PD/10%/60 s/Dry (**m**). Reprinted with permission from Ref. [3]. Copyright 2025, American Chemical Society.

Thummar et al. [3] fabricated a TFC OSN membrane by depositing a hydrophobic poly(methyl)siloxane (PMS) selective layer via a cross-linking reaction using the silicon-based cross-linking agent poly(methylhydrosiloxane) (PMHS) onto a hydrophilic PANMA porous support, where the structure and thickness of the PMS layer were controlled by using the support in a “wet state” (pores filled with water) or “dry state” (pores free of water). For the membrane prepared using the dry support, silicon is distributed within the pore structure, forming a denser silicon-based network (Figure 12h,i,k,l). After PMS coating, the water contact angle of the PANMA support layer increased from 108.5° to 113° (Figure 12g), demonstrating enhanced hydrophobicity. For the non-polar soybean-oil/hexane system, the optimal membrane (PD/10%/60s/Dry) achieved a flux of 31.8 L m^−2^ h^−1^ MPa^−1^ and an oil rejection of 93% at 1.38 MPa.

However, for silicon-containing membranes, the introduction of silicon-based moieties (e.g., PDMS segments or silica nanoparticles) significantly alters the balance between swelling resistance, cross-linking degree, and solvent permeance. Compared with conventional polymeric membranes without silicon modification, silicon-integrated membranes typically exhibit markedly enhanced resistance to swelling in non-polar solvents, but this improvement in structural stability often comes at the cost of reduced solvent permeance. For example, Thummar et al. [3] reported that a tightly cross-linked PDMS layer achieved an oil rejection of 93%, indicating strong resistance to solvent-induced swelling, but a moderate hexane permeance of 31.8 L m^−2^ h^−1^ MPa^−1^. This finding underscores a fundamental trade-off: while increased cross-linking enhances solvent resistance and selectivity, it can constrain free volume and limit throughput. Therefore, achieving an optimal balance between cross-linking density and membrane porosity is critical for maximizing both stability and performance.

### 3.4. Silicon-Based Nanofillers

To address the swelling effect of PDMS membranes, various fillers, especially silica nanoparticles, have been incorporated into the PDMS matrix to enhance the cross-linking density of the polymer network. This approach reduces membrane swelling in non-polar media and enables the fabrication of PDMS-based composite membranes to maintain high flux with improved selectivity [71,72,73].

Silica nanoparticles, most commonly fabricated via the sol–gel method (exemplified by the Stöber process), are synthesized through the controlled hydrolysis and condensation of alkoxide precursors like TEOS. This method enables the production of nanoparticles with excellent monodispersity, tunable size, and, crucially, a high specific surface area rich in hydroxyl groups. These features directly promote strong interfacial interactions with polymer matrices, which significantly enhances the mechanical strength and separation precision of the resulting composite material [72,73].

**Figure 13:**
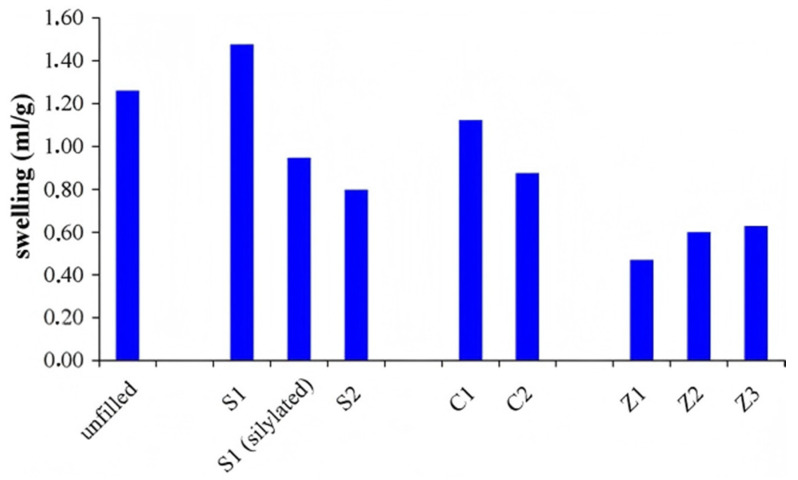
Swelling of PDMS filled with different fillers (toluene, RT). S1: 15 wt%, S2: 10 wt%; C1 and C2: 25 wt%; Z1, Z2 and Z3: 30 wt%. Reprinted with permission from Ref. [74]. Copyright 2006, Elsevier.

For instance, Gevers et al. [74] incorporated a variety of nanofillers, such as zeolite (e.g., ZSM-5 and USY) nanofillers and carbon nanofillers, into the PDMS matrix to address the swelling issues in non-polar solvent systems. The swelling rate of the PDMS membrane was reduced far more by zeolite incorporation than by silica and carbon materials, as shown in Figure 13. The prepared ZSM-5 filled PDMS membrane exhibits a toluene permeance of 5.8 L m^−2^ h^−1^ MPa^−1^ for the non-polar Wilkinson catalyst (925 Da)/toluene system, and its rejection increases from 78% to 98.5% as compared with the zeolite-free membrane. Meanwhile, it maintained 98% and 88% rejection at temperatures of 50 °C and 80 °C, respectively, demonstrating its excellent thermal and solvent stability.

**Scheme 2:**
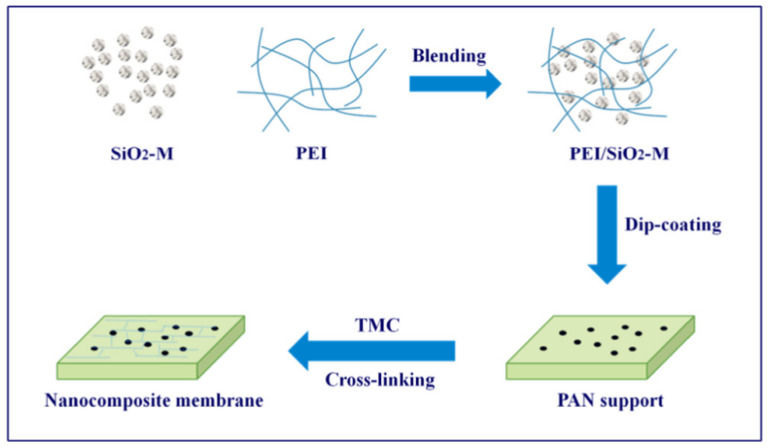
Preparation procedure of PAN/PEI SiO_2_−M. Reprinted with permission from Ref. [75]. Copyright 2016, Elsevier.

Li et al. [75] synthesized functionalized SiO_2_-M nanospheres (surface grafted with three functional groups: -C_6_H_5_, -SO_3_H, and-Py) via distillation-precipitation polymerization, and fabricated PAN/PEI-SiO_2_-M membranes through a dip-coating/interfacial polymerization method (Scheme 2). The resulting PAN/PEI-SiO_2_-C_6_H_6_ membrane exhibits a rejection of ≥92.8% for the polystyrene (PS 1000)/toluene system with a toluene flux of 12.8 L m^−2^ h^−1^ MPa^−1^.

**Figure 14:**
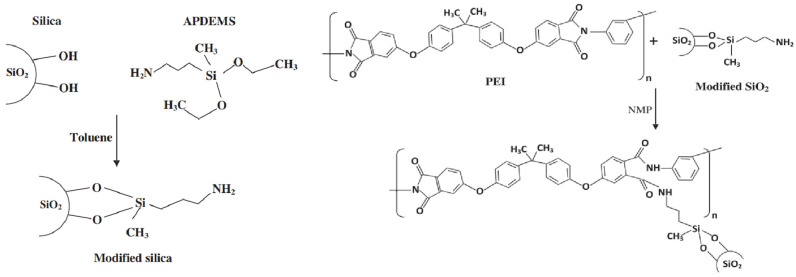
Schematic of amino-functionalization of nano silica using a silane coupling agent (APDEMS). Schematic of reaction between amino-functionalized silica nanoparticles and PEI. Reprinted with permission from Ref. [76]. Copyright 2013, Elsevier.

Silicon-based nanofillers can address the swelling effect of not only PDMS membranes but also polyamide membranes. For example, Namvar Mahboub et al. [76] fabricated a nano thin-film composite (n-TFC) membrane through interfacial polymerization of m-phenylenediamine (MPD) and trimesoyl chloride (TMC) on an amino-functionalized silica/polyetherimide (PEI) nanocomposite support layer (Figure 14). Under 1.5 MPa pressure, the optimal membrane (n-TFC1 with 5 wt% SiO_2_) delivered a rejection of 94.72% for dewaxed oil (MW ≈ 560 g mol^−1^) and a permeate flux of 10.41 L m^−2^ h^−1^ for a methyl ethyl ketone/toluene mixture solvent. In addition, the swelling degree of this optimal membrane in MEK decreased to 1.27 mL/g (a reduction of approximately 49%), and in toluene it decreased to 1.79 mL/g (a reduction of approximately 66%) (Table 4).

### 3.5. Summary of Performances of Silicon-Containing OSN Membrane

The performance of silicon-containing thin-film composite OSN membranes is summarized in Table 5, which provides a systematic comparison of key parameters—including silicon content, swelling behavior, solvent permeance, and solute rejection—across various silicon-integrated membranes. For membranes incorporating silicon fillers or cross-linkers, separation performance is strongly governed by size sieving and swelling suppression. The formation of Si-O-Si networks provides mechanical and structural stability, while hydrophobic surface characteristics enhance non-polar solvent permeation via favorable solubility-diffusion interactions.

## 4. Synergistic Fluorosilicone-Based OSN Membrane

Fluorosilicone-synergistic composite membranes have been a popular research area in the field of OSN in recent years. The high electronegativity and low polarizability of fluorine impart extremely low surface energy and strong solvent-repellent properties [77], while siloxane segments contribute essential flexibility and film-forming ability [78]. By combining the high stability of C-F bonds with the flexibility of siloxane chains, these membranes exhibit excellent solvent resistance and mechanical robustness [79,80] in non-polar solvent environments over extended periods—addressing a critical limitation of conventional membrane materials which often swell in organic solvents.

Specifically, researchers have adopted various fabrication strategies to incorporate fluorine and silicon components co-currently into the membrane structure, achieving significant performance enhancements. Typically, these membranes are fabricated through interfacial polymerization, surface grafting, blending modification, or surface coating to form ultra-refined separation layers with tailored structures and functionalities.

Introducing fluorine and silicon components co-currently via interfacial polymerization has proven to be an effective strategy. For example, Jimenez Solomon et al. [47] introduced fluorine (F)- and silicon (Si)-containing hydrophobic groups to fabricate organic solvent nanofiltration (OSN) membranes. They developed a novel, high-flux hydrophobic TFC membrane via interfacial polymerization between MPD and a mixture of fluorinated monoacyl chloride and TMC on a cross-linked polyimide ultrafiltration support surface, followed by surface modification to graft an amino-containing organosilicon monomer (polydimethylsiloxane co-3-aminopropylmethylsiloxane) onto the residual acyl chloride groups, as shown in Routine 9 presented in Figure 4. This kind of second-generation silicon-modified membrane exhibited extremely high toluene permeance of 115 L m^−2^ h^−1^ (30 bar), together with a rejection of 90% for polystyrene (MW ≈ 1000). Compared with the silicon/fluorine-free OSN membranes, the resultant OSN membranes exhibited remarkably enhanced anti-swelling performance, along with a significant increase in permeance toward toluene.

In addition to constructing separation layers through interfacial polymerization, surface activation and grafting modification of substrate membranes can also successfully introduce fluorosilane components, thereby optimizing the membrane’s surface characteristics and permeation performance. For example, Hugo et al. [81] used commercially available PVDF and PTFE membranes as substrates and used RF plasma-oxygen treatment to generate active surface groups so as to graft fluorinated silane (PDTS) on their surface in an acidic alcohol medium. This modification significantly improved the hydrophobicity, with water contact angles of 145.7° for PVDF and 150.5° for PTFE. Correspondingly, the permeate fluxes increased by 25% and 8.9% for PVDF and PTFE, respectively, together with a salt rejection of ≈99.99% for both membranes.

Furthermore, introducing siloxane as an additive into the membrane matrix through blending modification represents another effective strategy for constructing a fluorosilicone synergistic effect. Jing et al. [63] incorporated a two-dimensional siloxane additive into a PVDF nanofiltration membrane for the first time, producing a PVDF/siloxene mixed-matrix membrane (MMM) by the phase inversion method. The best performing variant, PVSI-075 (0.075 wt% siloxane), showed a pure water flux of 220 L m^−2^ h^−1^ MPa^−1^, MWCO of ~535 Da, and n-hexane flux of 110 L m^−2^ h^−1^ MPa^−1^. The siloxane induced tighter packing of PVDF molecular chains, resulting in a denser structure with reduced free volume and high orderliness, while preserving the hydrophobicity and chemical stability of PVDF.

**Figure 15:**
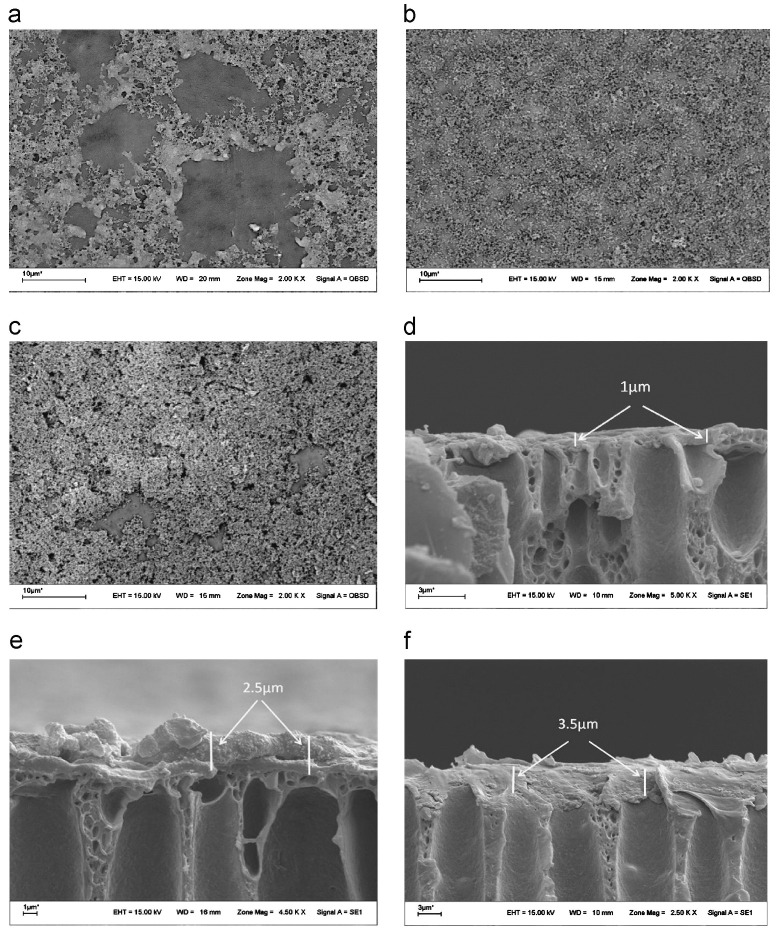
Surface and cross-section SEM images: (**a**,**d**) PVDF–10Si; (**b**,**e**) PVDF–12Si; and (**c**,**f**) PVDF–15Si. Reprinted with permission from Ref. [82]. Copyright 2013, Elsevier.

In contrast to the blending strategy, which involves uniformly doping functional components into the membrane matrix, the surface coating or casting method focuses on constructing an independent and structurally controllable separation functional layer on a prefabricated support. Li et al. [2] prepared a composite OSN membrane by solution casting fluorinated copolymer polydimethylsiloxane-polytrifluoropropylmethylsiloxane (PDMS-PTFPMS) on a PVDF support. The optimal OSN membrane (50% PTFPMS) showed optimal performance, with an n-hexane permeance of 30.61 L m^−2^ h^−1^ MPa^−1^ and a soybean oil rejection exceeding 95% during a 32-day stability test at 2.4 MPa, demonstrating its excellent solvent resistance and structural integrity. Firman et al. [82] prepared a PVDF support layer via the phase inversion method and applied either a PDMS or cellulose acetate (CA) separation layer via a cross-linking coating to create membranes for separating soybean oil/n-hexane and free fatty acids (FFAs). The PVDF-12Si membrane (12% PDMS coating) exhibited a water contact angle of 116.9° ± 3.5°, substantially higher than that of the PVDF-CA membrane (58.1°). As the PDMS concentration increased, the contact angles increased from 102.8° to 118.2°, enhancing the hydrophobicity. The introduction of Si-CH_3_ groups (with a low surface energy of ~19.8 mN/m) increased wettability toward hexane. The surface of the PVDF-12Si membrane was smooth and free of visible pores or protrusions, with the PDMS coating fully infiltrating and leveling the surface depressions of the PVDF support, resulting in a dense and uniform selective barrier, as shown in the SEM image in Figure 15. Under 2.0 MPa 30 °C and 25% oil concentration, the PVDF-12Si membrane achieved a pure hexane permeance of 22.5 L m^−2^ h^−1^ MPa^−1^, an oil/hexane mixture permeate flux of 20.3 L m^−2^ h^−1^, and retention rates of 80% for oil and 58% for FFA (free fatty acid, MW: 250–300 g/mol). Pagliero et al. [83] developed an asymmetric PVDF support membrane using phase inversion and then constructed a PDMS or CA separation layer by coating, forming PVDF-Si and PVDF-CA composite membranes. The PVDF-Si membrane showed a water contact angle of 114 ± 8°, and exhibited good chemical and physical stability after long-term permeation experiments, with only slight swelling/shrinkage observed after 48 h exposure to hexane (slightly rolled). At 30 °C, 0.78 MPa, and 25% oil concentration, the PVDF-Si membrane achieved a permeation flux of 12 L m^−2^ h^−1^ for n-hexane and a sunflower seed oil rejection of 46.2%. Its combination of high hydrophobicity and structural stability rendered it suitable for the efficient separation of non-polar solvent systems.

**Figure 16:**
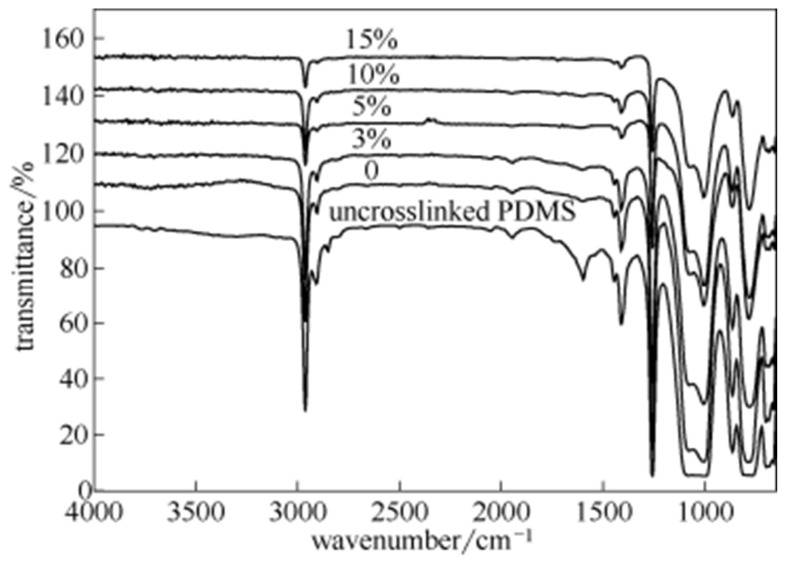
FT-IR spectra of cross-linked PDMS with different DNS-2 filling content. Reprinted with permission from Ref. [84]. Copyright 2015, Chemical Industry Press.

Nevertheless, despite the versatility and promising performance of these coating strategies, the PDMS-based separation layers remain susceptible to swelling in strongly non-polar solvents like hexane, which can compromise long-term stability and selectivity. To address this issue, Cai et al. [84] developed a DNS-2-filled PDMS/PVDF composite membrane by using a fluorinated PVDF support and incorporating hydrophobic, nanoscale white carbon black filler in the PDMS matrix. As presented in Table 6, the contact angle of the PDMS layer gradually increased from 115.9° for the unfilled membrane to progressively higher values with increasing filler content. Additionally, FT-IR analysis in Figure 16 reveals that the addition of white carbon black promoted PDMS cross-linking, reduced residual Si–OH groups, and enhanced thermal stability and hydrophobicity. Under a pressure of 2.4 MPa, the PDMS/PVDF composite membrane with 10% DNS-2 achieved a 97% rejection for hexane, while maintaining a flux of 2.2 kg m^−2^ h^−1^ and operational stability sustained over 13 days in non-polar solvents.

In summary, the diverse fabrication strategies and their corresponding performance outcomes collectively highlight the fundamental advantages of the fluorosilicone synergistic OSN membrane system. Compared with purely fluorinated or silicon-based membranes, fluorosilicone composite membranes synergistically integrate the benefits of both the rigid fluorinated segments contribute to effective size sieving and solvent selectivity, while the flexible siloxane network mitigates swelling. The low surface energy imparted by fluorine further enhances differential solute–solvent solubility and diffusion rates, leading to improved overall separation performance.

## 5. Separation Mechanism of OSN Technology in Non-Polar Solvent Systems

From the early stages of membrane technology, researchers have investigated the mass transfer mechanism in membrane processes. Understanding mass transfer and separation mechanisms is essential for guiding the design and regulation of membrane structures, as well as for predicting membrane performance. Generally, the permeation and selectivity of OSN membranes under different solvent conditions are primarily influenced by the surface properties of the membrane (e.g., hydrophilicity/hydrophobicity, membrane pore size) and membrane–solvent–solute interactions [85]. The key separation mechanisms of OSN technology include size sieving, electrostatic repulsion, and dissolution–diffusion effects.

### 5.1. Size Screening and Pore Control

Size screening is a separation mechanism based on physical exclusion, wherein a network of nanoscale pores or free volume cavities within the membrane allows selective transport. Under an applied pressure, molecules smaller than the membrane’s pore size—typically solvent molecules—can permeate, while larger molecules, such as macromolecular solutes, are rejected. This process is governed primarily by the size and shape compatibility between solute molecules and the membrane’s pore structure, rather than their charge, chemical nature, or specific interactions with the membrane material.

Effective strategies to tailor membrane pore size for enhanced size-sieving performance include modulation of cross-linking degree and the introduction of rigid porous nanofillers. Si et al. [86] introduced fluorinated monomers that not only impart hydrophobicity but also affect the cross-linking density of PDMS networks through their rigid molecular structure, thereby regulating the transport channel dimensions. Similarly, Pang et al. [87] embedded the rigid nanofiller ZIF-8 into the membrane, enabling precise control over pore architecture and facilitating size-selective molecular separation.

### 5.2. Hydrophobic Interaction and Membrane Swelling

The OSN technology in non-polar solvent systems presents critical challenges such as membrane material swelling, reduced selectivity, and diminished stability. Fluorine- and silicon-based materials offer excellent solvent resistance and separation performance due to their unique chemical structures. Hydrophobic interaction refers to the thermodynamic tendency of hydrophobic groups (e.g., -CF_3_, -CF_2_-, Si-CH_3_ in fluorine- and silicon-based materials) to minimize contact with polar molecules, while exhibiting affinity toward non-polar solvents [88]. In OSN membranes, this interaction determines the wettability of the membrane surface by non-polar solvents and the compatibility between the membrane matrix and solvents, directly affecting solvent permeation efficiency and membrane structural integrity. Hydrophobic interaction can be quantitatively expressed as a solubility parameter, which also affects the membrane swelling.

According to Hildebrand’s solubility parameter theory [89], the swelling behavior of a polymer in non-polar solvents is largely governed by the degree matching between the solubility parameters (δ) of the solvent and that of the membrane material. When the δ values of the solvent and polymer are closely matched, the polymer may absorb solvent and swell significantly. For example, when n-hexane (δ ≈ 14.9 MPa^1/2^) contacts PDMS (δ = 15 MPa^1/2^), the polymer network can expand, resulting in high permeation of n-hexane yet poor selectivity [84,90]. On the other hand, when the difference between the solubility parameters of the membrane and the solvent (∆δ = ∣δ_p_ − δ_s_∣) is maintained below around 3 MPa^1/2^ (where δ_p_ is the membrane solubility parameter and δ_s_ is the solvent solubility parameter), solvent permeation can be achieved while avoiding excessive swelling [91].

Fluorosilicone synergistic composite membranes enable precise control over mass transfer kinetics in non-polar solvent environments by aligning solubility parameters and optimizing diffusion pathways. Non-polar solvents typically have solubility parameter values (δ_s_) in the range of ~14–30 MPa^1/2^ (e.g., toluene ≈ 18.2 MPa^1/2^, cyclohexane 16.8 MPa^1/2^). By contrast, fluorine- and silicon-based compounds facilitate tailoring the membrane solubility parameters (δ_p_) to ~13–16 MPa^1/2^ via compositional tuning. For example, PTFE (δ_p_ ≈ 12.7 MPa^1/2^) lowers the polymer δ_p_ relative to polymers such as polysulfone (δ_p_ ≈ 21 MPa^1/2^) thanks to low-polarity C–F bonds, while PDMS (δ_p_ ≈ 14.9 MPa^1/2^) offers balance between δ_p_ and permeance due to its flexible Si-O-Si backbone. In a study by Caiet et al. [84], incorporation of hydrophobic white carbon black (SiO_2_) nanoparticles in PDMS was shown to reduce swelling of a PVDF base film: surface silanol groups on SiO_2_ reacted with terminal PDMS hydroxyls to form a “point–line” anti-swelling network. This strategy adheres to the “like-dissolves-like” principle: when the δ distance (∆δ = |δ_p_ − δ_s_|) is too large, solvent permeance is compromised, and when ∆δ is too small excessive swelling occurs. Achieving an optimal ∆δ thus enables a balance between low swelling and high permeance [26,92].

## 6. Application in Homogeneous Catalyst Recovery

In the chemical industry, catalytic systems are indispensable for optimizing production processes. Numerous studies have demonstrated that the introduction of catalysts can significantly improve the conversion efficiency of reactants and the selectivity of target products [93], while simultaneously reducing production costs and improving the environmental friendliness of industrial operations [94]. Among these systems, homogeneous organometallic catalysts hold particular importance due to their high activity and versatility in fine chemical synthesis. For example, homogeneous palladium-catalyzed coupling reactions account for 22% of all reactions in the pharmaceutical industry [95,96]. However, the widespread use of precious metal catalysts such as palladium and rhodium presents critical challenges arising from their high cost and potential toxicity. Stringent pharmaceutical regulations require the removal of residual metals from final products to ensure safety and compliance, making the separation and recovery of homogeneous catalysts a key issue in industrial-scale applications. Traditional separation methods (e.g., distillation, adsorption, or precipitation) are often energy-intensive, inefficient and may lead to catalyst deactivation or secondary waste generation. OSN, as a non-thermal, pressure-driven separation technology, provides a promising and non-destructive alternative for recovery of homogeneous catalysts. Its advantages of low energy consumption, mild operating conditions, and effective molecular-level selectivity make it an attractive strategy for sustainable catalyst recycling and process intensification.

### 6.1. Homogeneous Catalyst Recovery Based on OSN

Currently, commonly used homogeneous metal catalysts in industry include palladium (Pd)-, ruthenium (Ru)-, rhodium (Rh)-, and gold (Au)-based complexes [95].

(1) Pd-based homogeneous catalyst

**Figure 17:**
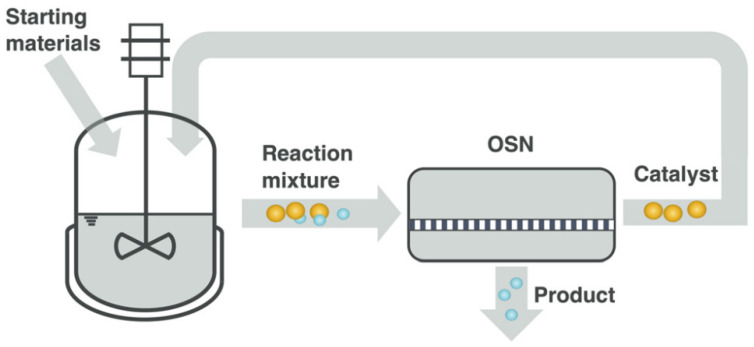
Schematic of the catalyst recovery and reuse: the recovered catalyst/ligand is added to the same quantity of starting materials as in the first cycle for further reactions and subsequent OSN diafiltration. Reprinted with permission from Ref. [98]. Copyright 2025, Royal Society of Chemistry.

Datta et al. [97] deposited a 10% PDMS coating on a PAN support to fabricate a PDMS/PAN composite membrane for catalyst recovery in non-polar solvent systems. The resulting membrane exhibited an excellent rejection of 99.95% for the Pd-based catalyst solute Pd(PhCN)_2_Cl_2_ (MW > 5000 Da) under 3.0 MPa and 50 °C, demonstrating its strong rejection capability for large organometallic solutes. Xiao et al. [98] used a PDMS-based Borsig NF-1 OSN membrane to recover a palladium catalyst from a non-polar 2-methyltetrahydrofuran (2-MeTHF) medium. This membrane contained a cross-linked Si-O-Si network that imparted highly hydrophobic. At an operating pressure of 2.0 MPa, the membrane achieved a Pd(dba)_2_ (bis(benzylideneacetone) palladium(0), M_n_ = 575 g mol^−1^) rejection of 98.0%, while maintaining a 2-MeTHF permeation flux of 39 L m^−2^ h^−1^ MPa^−1^. The recovered catalyst–ligand complex retained its catalytic activity and can be reused, as shown in Figure 17, enabling multiple reaction cycles without significant performance degradation.

(2) Ru-based homogeneous catalyst

Rabiller Baudry et al. [99,100] used a commercial STARMM™ 112 membrane to separate the Ru-based catalyst Grubbs Hoveyda II (M_n_ = 627 Da) from the non-polar solvent toluene. At 2.0 MPa and 40 °C, the catalyst rejection reached 99.5%. Roengpithya et al. [101] further evaluated the STARMM™ 122 for the separation of Ru-based catalysts Ru-Cymene and P1-t-Oct in toluene. Under 3.0 MPa, the corresponding catalyst retention rates were 92% and 99.6%, respectively, with a toluene permeation flux of toluene of approximately 40 L m^−2^ h^−1^ MPa^−1^. Schoeps et al. [102] prepared PDMS/PAN composite membranes to recover Grubbs II and Hoveyda-type Ru catalysts (M_n_ ≈ 1100 Da, containing benzylidene carbene ligands) from toluene. At 2.5 MPa and 30 °C, the rejection of the catalysts reached 99.8% for both Ru complexes and the toluene permeate flux was about 51 L m^−2^ h^−1^ MPa^−1^. Van der Gryp et al. [103] systematically investigated the separation of five Grubbs-type Ru catalysts using a STARMMTM™ OSN membrane series in a toluene/alkane solvent system. Among them, the STARMM^TM^ 228 membrane showed the best performance, yielding residual catalyst concentrations below 9 ppm and an almost complete (≈100%) retention. Under 3.0 MPa, the membrane exhibited fluxes of 9.2 kg m^−2^ h^−1^ for 1-octene and 0.67 kg m^−2^ h^−1^ for 7-tetradecene, with an overall product permeance of 12 L m^−2^ h^−1^ MPa^−1^.

(3) Rh-based homogeneous catalyst

Razak et al. [104] used a commercial Starmem 240 membrane to separate the Rh-based catalyst HRh(CO)(PPh_3_)_3_ (M_n_ = 918.78 Da, containing a triphenylphosphine ligand) from the non-polar solvent toluene. Under operating conditions of 2.5 MPa and 30 °C, the catalyst rejection reached 93%, and the toluene permeate flux was approximately 42 L m^−2^ h^−1^ MPa^−1^. Dreimann et al. [105] developed a series of PolyAn polymer membranes for the separation of Rh-based catalysts, Rh(acac)(CO)_2_-Xantphos (M_n_ = 258 + 579 Da) and Rh(acac)(CO)_2_-Bipephos (M_n_ = 258 + 787 Da), in the non-polar solvent systems. At 2.5 MPa, both catalysts exhibited high retention efficiencies of 97%. The corresponding toluene permeation flux was approximately 35 L m^−2^ h^−1^ MPa^−1^, confirming that these polymeric OSN membranes effectively balance catalyst rejection and solvent transport.

(4) Au-based homogeneous catalyst

Bayrakdar et al. [106,107] used a PDMS-based Borsig oNF-1 membrane to recover an Au-based catalyst from the non-polar solvent. When applied to the separation of the Au(OTf)(IPr) complex in THF, the membrane achieved a catalyst retention of 98.5% at 2.0 MPa, with a THF permeation flux of ~41 L m^−2^ h^−1^ MPa^−1^. Under similar conditions, the membrane was also used to separate the [Au_2_(L)Cl_2_] complex in mixed THF/2-MeTHF solvent systems, attaining an even higher retention of 99.5% and a corresponding permeation flux of about 38 L m^−2^ h^−1^ MPa^−1^ at 2.0 MPa.

**Figure 18:**
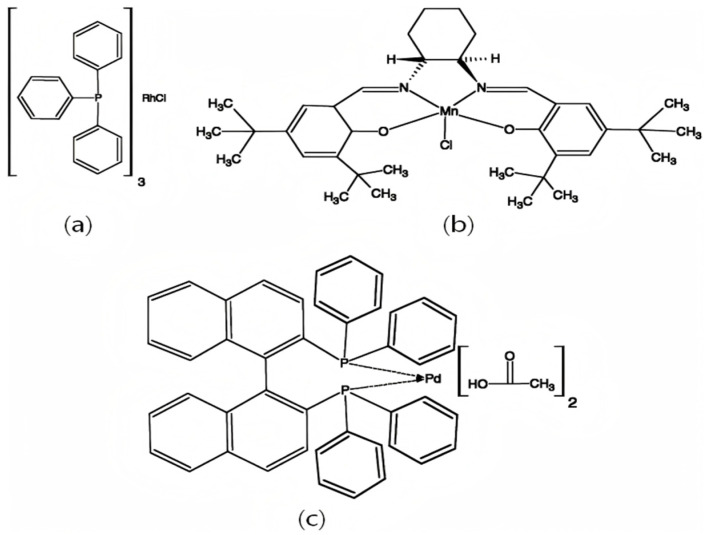
Catalyst structures: (**a**) the Wilkinson catalyst; (**b**) the Jacobsen catalyst; (**c**) Pd-BINAP. Reprinted with permission from Ref. [108]. Copyright 2002, Elsevier.

Scarpello et al. [108] conducted OSN studies on three representative homogeneous organometallic catalysts—Jacobsen, Wilkinson, and Pd BINAP catalysts—commonly used in commercial organic synthesis (as shown in Figure 18).

### 6.2. OSN Membrane Performance Under Non-Neutral and Harsh Chemical Conditions

While many reported catalyst recovery processes using OSN are carried out in neutral organic media (e.g., toluene and ethyl acetate), industrial catalytic processes usually involve acidic or alkaline environments that pose challenges to membrane stability. A number of studies have begun to evaluate OSN membrane performance under such extreme chemical conditions. Lee et al. [109] prepared cross-linked polybenzimidazole (PBI)-based OSN membranes, which exhibited excellent stability under strongly alkaline conditions (pH = 13), with separation performance recoverable after subsequent neutral solvent filtration, indicating reversible swelling behavior. Polyimide (PI) membranes, known for their inherent chemical robustness, have also been shown to maintain high performance in strong acidic and organic solvent environments [110]. In addition, the composite OSN membranes based on polyether ether ketone (PEEK) and graphene oxide (GO), as reported by Pang et al. [111], also showed long-term stability (180 days) in strong acids, strong alkalis, and various organic solvents.

Despite these advances, highly corrosive environments such as strong acids or alkalis may still hydrolyze the sensitive chemical bonds in membrane materials, ultimately degrading performance. As early as 1971, Deiasi et al. [112] found that the imide bonds in surface polyimide might undergo hydrolysis in aqueous environments with low pH values (especially pH < 2.0), thus resulting in deterioration of mechanical properties. Additional work on the surface modification of PI films also confirmed that acid–alkali treatment could alter both surface chemical composition and morphology [113]. At present, systematic studies on the long-term chemical stability of fluorosilicone-based OSN membranes across a broad pH range or in the presence of oxidants or other reactive species are still scarce. Further research is needed to define the operational boundaries of these membranes in complex, real-world catalytic systems and to develop design strategies for enhanced stability under harsh chemical conditions.

## 7. Challenges and Future Prospects

### 7.1. Challenges for Non-Polar System Separation

Fluorine- and silicon-based organic solvent nanofiltration (OSN) membranes show great potential for non-polar solvent separation due to their high hydrophobicity and chemical stability. However, their transition from lab to industry faces several core challenges.

First, material and preparation costs are high. The synthesis of fluorinated polymers and functionalized silica is complex, and key raw materials are often expensive. Preparation techniques like interfacial polymerization face difficulties, such as the poor solubility of fluorinated monomers, requiring costly additives.

Beyond cost constraints, ensuring long-term operational stability presents another major hurdle. Challenges here are prominent, encompassing mechanical stability, fouling management, and chemical recyclability. Long-term mechanical stability under continuous industrial conditions remains a critical bottleneck. Most studies report only short-term performance. For instance, Alduraiei et al. [26] reported stable operation in toluene for 5 days, while systematic data over hundreds of hours are scarce. Promisingly, Li et al. [2] developed a PDMS-PTFPMS/PVDF membrane that operated continuously in n-hexane for 32 days (≈768 h) without significant performance decline. Nevertheless, membrane evolution under combined solvent, pressure, and temperature stresses needs deeper study.

Alongside mechanical stability, the adaptability of membranes to fouling and cleaning protocols is equally critical. While fouling is less severe than in aqueous systems, solute adsorption can cause flux decline. Common cleaning methods include solvent flushing and backwashing. Xin et al. [114] used hot solvent forward flushing with backwashing to recover flux. Membranes with ultra-low surface energy, like the perfluoropolyether (PFPE)-coated membranes by Li et al. [60], allow simple n-hexane flushing for recovery. However, improper cleaning solvents can cause swelling or delamination. Future work should focus on understanding fouling mechanisms in non-polar media and developing compatible in situ cleaning strategies.

Furthermore, looking at the end-of-life phase, the chemical recyclability of these cross-linked membranes is largely unexplored. Most are designed for durability, not easy decomposition, posing challenges for end-of-life disposal or material recovery.

### 7.2. Future Prospects

Addressing these multifaceted challenges requires a coordinated research and development effort across several key directions.

In Material Innovation, developing low-cost, high-performance materials is fundamental. This includes synthesizing new fluorinated co-monomers and bio-based siloxanes to reduce costs. Fluorine-silicon synergy should be leveraged, combining the rigidity of C-F bonds with the flexibility of Si-O-Si bonds to create membranes balancing anti-swelling properties and high flux.

Building on advanced materials, Long-Term Performance Optimization is crucial. Enhancing anti-aging, reusability, and fouling resistance involves methods like tuning cross-linking density and applying anti-fouling coatings. Concurrently, standardized aging tests and predictive lifespan models must be established.

To close the sustainability loop, focused work on Recyclability and Sustainability is essential. This involves developing degradable fluorosilicone materials (e.g., with cleavable ester bonds) or using bio-based supports. End-of-life strategies, such as monomer recovery via de-cross-linking or filler recycling, should be explored to enable a circular economy.

Moreover, interdisciplinary approaches will be vital to accelerate progress. Artificial intelligence can accelerate material design by predicting structure–performance relationships, while molecular simulations can provide fundamental insights into mass transfer and aging mechanisms in non-polar solvents. With advancements in materials, processes, and characterization, fluorosilicone-based OSN technology is poised to overcome current barriers and enable large-scale applications in areas like catalyst recovery and petrochemical refining, supporting greener industrial processes.

## Figures and Tables

**Table 1:** Physiochemical properties of the selected organic solvents. Reprinted with permission from Ref. [59]. Copyright 2020, Elsevier.

Solvent	Polarity Index ^a^	Viscosity ^b^ (cP)	Molar Volume ^b^ (cm^3^/mol)	Hansen Solubility Parameter ^c^ δ_Tot_, (MPa^1/2^)
DMSO	7.2	1.996	71.3	26.6
DMF	6.4	0.8967	77.1	24.6
Toluene	2.4	0.5780	105.7	18.2
Acetone	5.1	0.3116	74.1	19.3
MeOH	5.1	0.5686	40.6	29.6
Ethyl acetate	4.4	0.4338	98.5	18.1
EtOH	4.3	1.017	58.2	25.0
Hexane	0.1	0.3071	130.5	14.9

^a^ Polarity indexes. ^b^ Viscosity and density are obtained from Aspen HYSYS software at 22 °C and 0.5 MPa. Molar volume is calculated based on density and molecular weight. ^c^ Hansen solubility parameters are obtained from HSPiP software. Teflon AF2400’s Hansen solubility parameter δ_Tot_: 16.1 MPa^1/2^.

**Table 2:** Performances of fluorinated thin-film composite OSN membrane.

Membrane	Treatment Methods	Solute (MW, g mol^−1^)	Solvent	Solvent Permeance (L m^−2^ h^−1^ MPa^−1^)	Rejection (%)	Year and Reference
TFMPD-TMC/HFBC on PAN substrates	IP	Hexaphenyl-	Toluene	100	87	2021, [26]
		Benzene (534)				
OTFA-TMC on PK substrates	IP	Irganox (3114)	Toluene	50	96	2024, [25]
TAPB-TFPA on PAN substrates	IP	CR (687)	Toluene	110	>95	2022, [48]
MPD/5-TFMPD (or 4-TFOPD)-TMC on	IP	Jacobsen catalyst (635)	ethyl acetate	209.4 or 117	98.2 or 98.8	2024, [51]
PI UF [49] substrates						
PDMS-PTFPMC on PVDF substrates	Casting	soybean oil (283)	n-Hexane	30.6	96	2017, [2]
PVDF	XDA cross- linked	RB (1017)	Toluene	9.4	98	2018, [44]
AF2400/PTFE	_____	soybean oil (283)	n-Hexane	8–11	>98	2017, [40]
Teflon AF2400 on PE substrates	Coating	Triglyceride (639)	n-Hexane	14.5	99.5	2020, [59]
MWCNT/PVDF on PTFE substrates	Cohering	Water (18)	n-Octane	651.4 *	99.87	2022, [62]
PEPE on Matrimid PI substrates	Coating	β-carotene (536.87)	n-Hexane	7.7	94.2	2019, [60]

* Calculated value.

**Table 3:** Summary of fluorine functional group and surface energy in fluorinated membranes.

Membrane	Fluorinated Functional Group	Surface Energy Change (Water Contact Angle /Surface Energy)	Year and Reference
BPAF-TMC on PAN substrates	-CF_3_ (BPAF monomer)	WCA: 87.1°	2023, [38]
PVDF/PLA (Electrospinning blend loaded with Ag/CuO NPs)	-CF_3_ + -CF_2_- (PVDF/PLA)	WCA > 150°	2023, [27]
TFMPD-TMC/HFBC on PAN substrates	-CF(CF_3_)_2_: HFBC -CF_3_: TFMPD	WCA ≈ 100°	2021, [26]
PFPE (Fluorolink AD1700) coated on HDA-cross-linked Matrimid	-CF_2_O-, -CF_2_CF_2_O-(PFPE)	WCA: 124°	2019, [60]
PVDF/siloxene mixed matrix membrane	-CF_2_- (PVDF)	WCA: 85°	2021, [63]
CMS-7 on ePTFE	-CF_2_-, -O-CF_2_-CF_2_-O-	WCA > 90°	2018, [64]
PDMS-PTFPMS on PVDF	-CF_3_ (PTFPMS)	WCA: 120°	2017, [2]
AF2400 on PTFE	-CF_2_-, -O-CF_2_-CF_2_-O-	WCA: 114.8°	2017, [40]
OTFA-TMC on PK substrates	-CF_3_ (OTFA monomer)	Surface Energy: 47.4 mN/m	2024, [25]

**Table 4:** Effect of amino-functionalized silica amount on the chemical and mechanical stabilities of nanocomposite membranes. Reprinted with permission from Ref. [76]. Copyright 2013, Elsevier.

Sample	Swelling Degree (mL/g) in MEK	Swelling Degree (mL/g) in Toluene	Tensile Strength at Break (MPa)
PEI	2.49 ± 0.07	5.27 ± 0.1	8.56 ± 0.43
PEI-5	1.27 ± 0.11	1.79 ± 0.14	12.64 ± 0.24
PEI-10	1.83 ± 0.02	2.57 ± 0.02	10.65 ± 0.12
PEI-15	2.13 ± 0.09	3.76 ± 0.6	7.29 ± 0.41
PEI-20	2.24 ± 0.03	4.90 ± 0.47	5.91 ± 0.63

**Table 5:** Performances of silicon-containing thin-film composite OSN membranes.

Membrane	Treatment Methods	Silicon Content	Swelling Degree (Δ%)	Solute (MW, g mol^−1^)	Solvent	Solvent Permeance (L m^−2^ h^−1^ MPa^−1^)	Rejection (%)	Year and Reference
PDMS substrates	ZSM-5 Filling	30 wt% (ZSM-5)	——	Wilkinson catalyst (925)	toluene	5.8	98.5	2006, [74]
SiO_2_-C_6_H_6_ on the PAN/PEI substrates	IP and cross- linking	3.0 wt% (SiO_2_-C_6_H_6_)	——	Polystyrene (1000)	toluene n-heptane	12.8 21.7	92.8	2016, [75]
PMS on the PANMA substrates (Dry and Wet)	coating	PD/10%/60s/ Wet: 25 at% Si; PD/10%/60s/ Dry: 26 at% Si	——	soybean oil (900)	n-hexane	31.8 105.7	93 75	2025, [3]
PDMS-PTFPMS on the PVDF substrates	casting	15.61 at% Si	197.1% → 39.2% (PDMS → F50-M)	soybean oil (900)	n-hexane	30.6	>95	2017, [2]
PEG/MPD-TMC/PDMS on PAN substrates	IP	1.24 wt% Si (EDX, PSf-TFC _PEG/PDMS-0.03_)	——	PEG (600)	toluene	7.2	70~84	2021, [22]
n-TFC1 membrane with amino-functionalized SiO_2_	IP	5 wt% SiO_2_	MEK: 2.49 → 1.27 mL/g; toluene: 5.27 → 1.79 mL/g	dewaxed oil (~560)	MEK/ toluene	6.94	94.72	2013, [76]

**Table 6:** Contact angles of PDMS with different DNS-2 filling content. Reprinted with permission from Ref. [84]. Copyright 2015, Chemical Industry Press.

DNS-2 Filling Content/%	Contact Angle/(°)
0	115.9 ± 0.3
3	117.6 ± 0.5
5	118.7 ± 0.4
10	118.9 ± 0.7
15	124.0 ± 1.8

## Data Availability

No new data were created or analyzed in this study. Data sharing is not applicable to this article.
